# Supplementary material for: Fiber microstructure quantile (FMQ) regression: A novel statistical approach for analyzing white matter bundles from periphery to core
Source: Imaging Neurosci (Camb). 2025 May 7;3:imag_a_00569. doi: 10.1162/imag_a_00569 (PMC12319761; doi:10.1162/imag_a_00569)
Supplement: Supplementary Material [file imag_a_00569-supp.pdf]

Supplementary Materials for "Fiber Microstructure Quantile (FMQ) Regression: A Novel Statistical Approach for Analyzing White Matter Bundles from Periphery to Core"

S1. Investigation of Mean Diffusivity (MD)

In this section, we provide a white matter analysis based on an investigation of the microstructure measure MD. The regression using the mean of this data is named MD Mean Regression.

We first present the mean squared errors (MSEs) obtained from regression models assessing the associations between white matter tracts and a scalar factor across multiple fiber bundles (Table S1). The MSE serves as a measure of the overall model fit, with lower values indicating better alignment between the model estimates and the observed data. The FMQ regression consistently produces the lowest MSEs compared to both MD Mean Regression and AFQ Regression models. Similar to our results when studying FA, this finding suggests that the FMQ regression method provides a superior model fit, capturing the relationship between the scalar factor and white matter tracts more effectively than the other approaches.

Next, we present visualizations of the bundle experiments (Figures S1-S8) that investigate MD. Similar to the results when studying FA, we identify multiple significant associations, including findings unique to males or females. Significant findings when investigating MD using the proposed FMQ regression are in line with the results from the MD mean regression.

Table S1. MSEs for MD Mean Regression, AFQ Regression, and FMQ Regression models. Each value represents the MSE, with standard errors provided in parentheses for AFQ and FMQ regressions. Lower MSE values indicate better model fit, with FMQ regression consistently showing the lowest MSEs among the three methods, suggesting superior model performance in capturing the associations between white matter tracts and the scalar factor.

Fiber Bundle	Scalar Factor	MD Mean Regression	AFQ Regression	FMQ Regression
AF left	PicVocab	4.61e-09	1.37e-08 (9.11e-09)	**5.12e-10 (3.84e-10)**
	ReadEng	4.59e-09	1.37e-08 (9.14e-09)	**5.21e-10 (3.94e-10)**
AF Right	PicVocab	4.83e-09	2.72e-08 (1.45e-08)	**5.05e-10 (4.68e-10)**

	ReadEng	4.84e-09	2.72e-08<br>(1.45e-08)	**5.10e-10**<br>**(4.77e-10)**
UF Left	ListSort	3.14e-09	9.75e-09<br>(4.26e-09)	**2.00e-10**<br>**(5.61e-11)**
	PicSeq	3.16e-09	9.74e-09<br>(4.24e-09)	**2.00e-10**<br>**(6.53e-11)**
UF Right	ListSort	3.45e-09	1.07e-08<br>(5.27e-09)	**2.47e-10**<br>**(8.93e-11)**
	PicSeq	3.49e-09	1.07e-08<br>(5.26e-09)	**2.73e-10**<br>**(1.10e-10)**
CST Left	Endurance	2.23e-09	2.80e-08<br>(1.72e-08)	**2.79e-10**<br>**(3.30e-10)**
	GaitSpeed	2.23e-09	2.80e-08<br>(1.72e-08)	**2.82e-10**<br>**(3.36e-10)**
CST Right	Endurance	2.31e-09	2.97e-08<br>(2.09e-08)	**3.32e-10**<br>**(3.37e-10)**
	GaitSpeed	2.31e-09	2.98e-08<br>(2.10e-08)	**3.26e-10**<br>**(3.49e-10)**
CB Left	CardSort	3.61e-09	9.73e-09<br>(4.76e-09)	**2.20e-10**<br>**(2.76e-10)**
	Flanker	3.59e-09	9.75e-09<br>(4.77e-09)	**2.15e-10**<br>**(2.80e-10)**
CB Right	CardSort	2.93e-09	7.17e-09<br>(3.17e-09)	**6.79e-11**<br>**(3.18e-11)**
	Flanker	2.92e-09	7.16e-09<br>(3.16e-09)	**6.62e-11**<br>**(3.22e-11)**

Table S2. The statistical significance of regression coefficients related to the scalar factors for all the associations that we investigate. An association where there is at least one significant regression coefficient will be labeled with an asterisk.

Figure	Fiber Tract	Neuro-behavioral Function	Scalar Factor	Sex	MD Mean Regression		AFQ Regression		FMQ Regression	
					LHem	RHem	LHem	RHem	LHem	RHem
Figures S1-S2	Arcuate Fasciculus (AF)	Language	PicVocab	Female			*			
				Male	*				*	
			ReadEng	Female	*		*		*	
				Male						
Figures S3-S4	Uncinate Fasciculus (UF)	Memory	PicSort	Female		*				*
				Male					*	
			ListSort	Female						
				Male		*				
Figures S5-S6	Cortico-spinal Tract (CST)	Motor	Endurance	Female					*	*
				Male			*		*	
			GaitSpeed	Female						
				Male						
Figures S7-S8	Cingulum Bundle (CB)	Executive function	CardSort	Female						
				Male						
			Flanker	Female						
				Male						

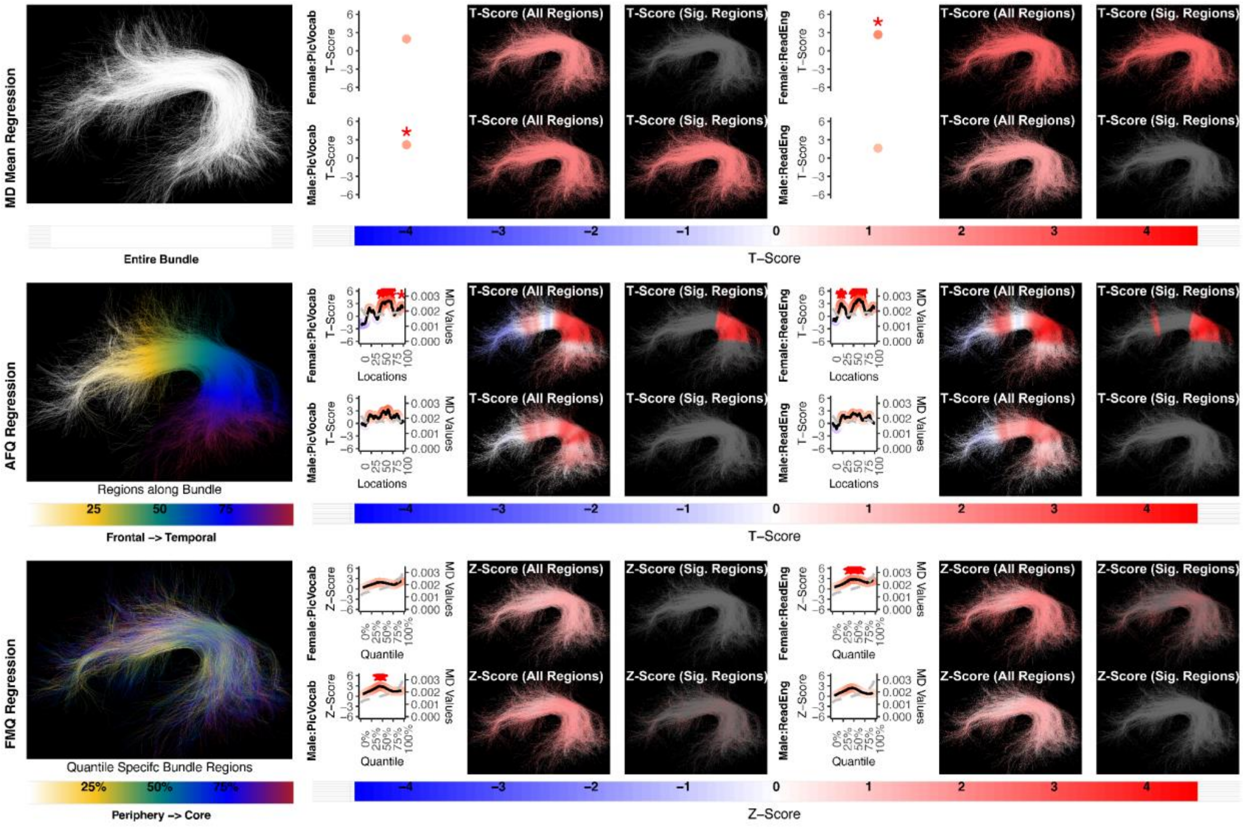

Figure S1. The association between AF left MD and language performance. For each experiment, plots of Z- or T-scores (solid line) and FA (dashed line) are provided, with red asterisks indicating BH-FDR-corrected statistical significance. Visualizations of Z- and T-scores are provided.

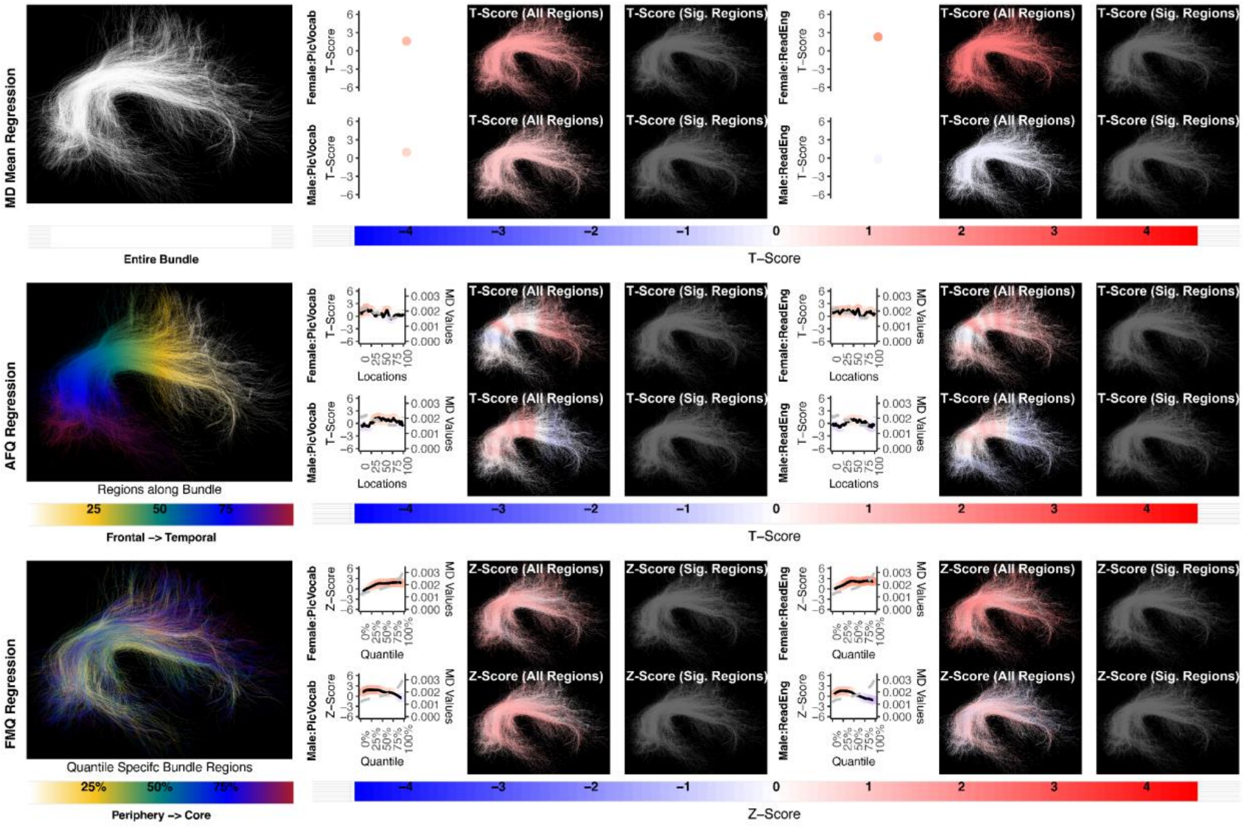

Figure S2. The association between AF right MD and language performance. For each experiment, plots of Z- or T-scores (solid line) and FA (dashed line) are provided, with red asterisks indicating BH-FDR-corrected statistical significance. Visualizations of Z- and T-scores are provided.

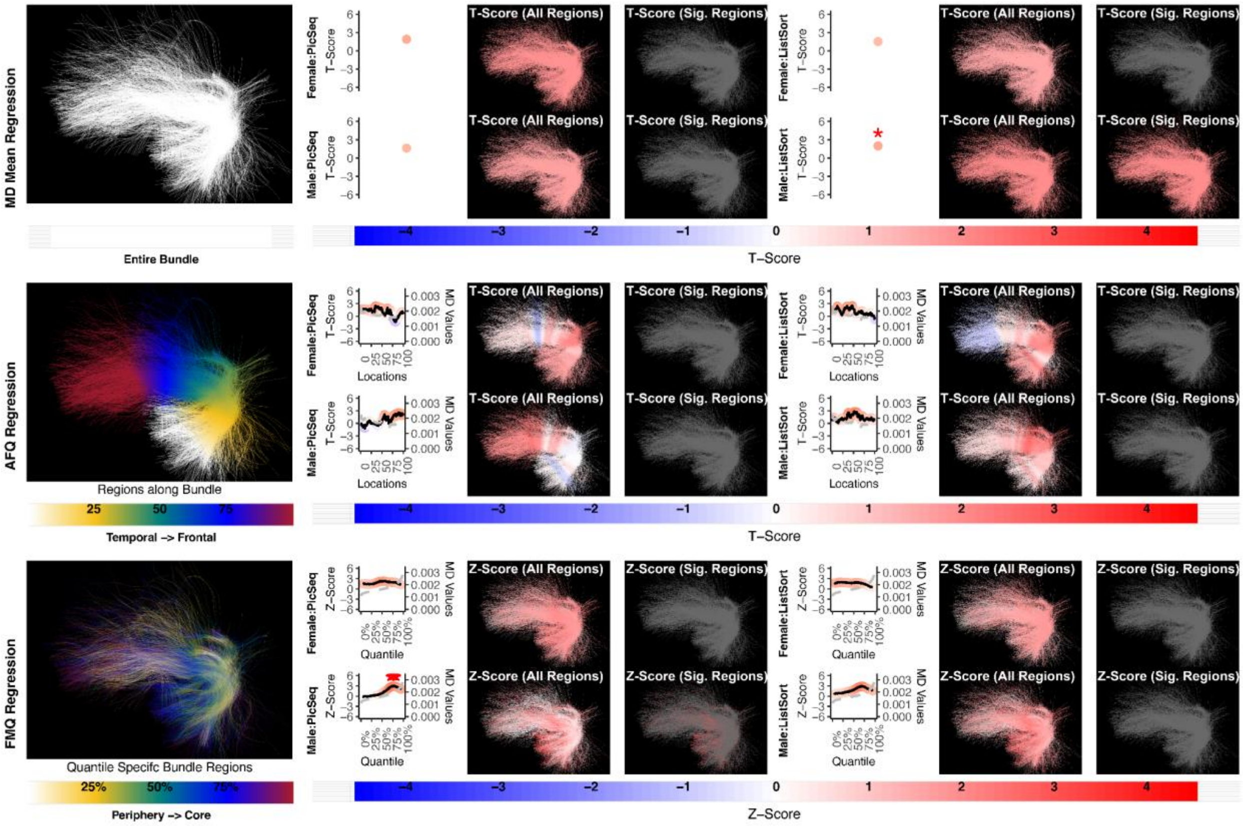

Figure S3. The association between UF left MD and memory performance. For each experiment, plots of Z- or T-scores (solid line) and FA (dashed line) are provided, with red asterisks indicating BH-FDR-corrected statistical significance. Visualizations of Z- and T-scores are provided.

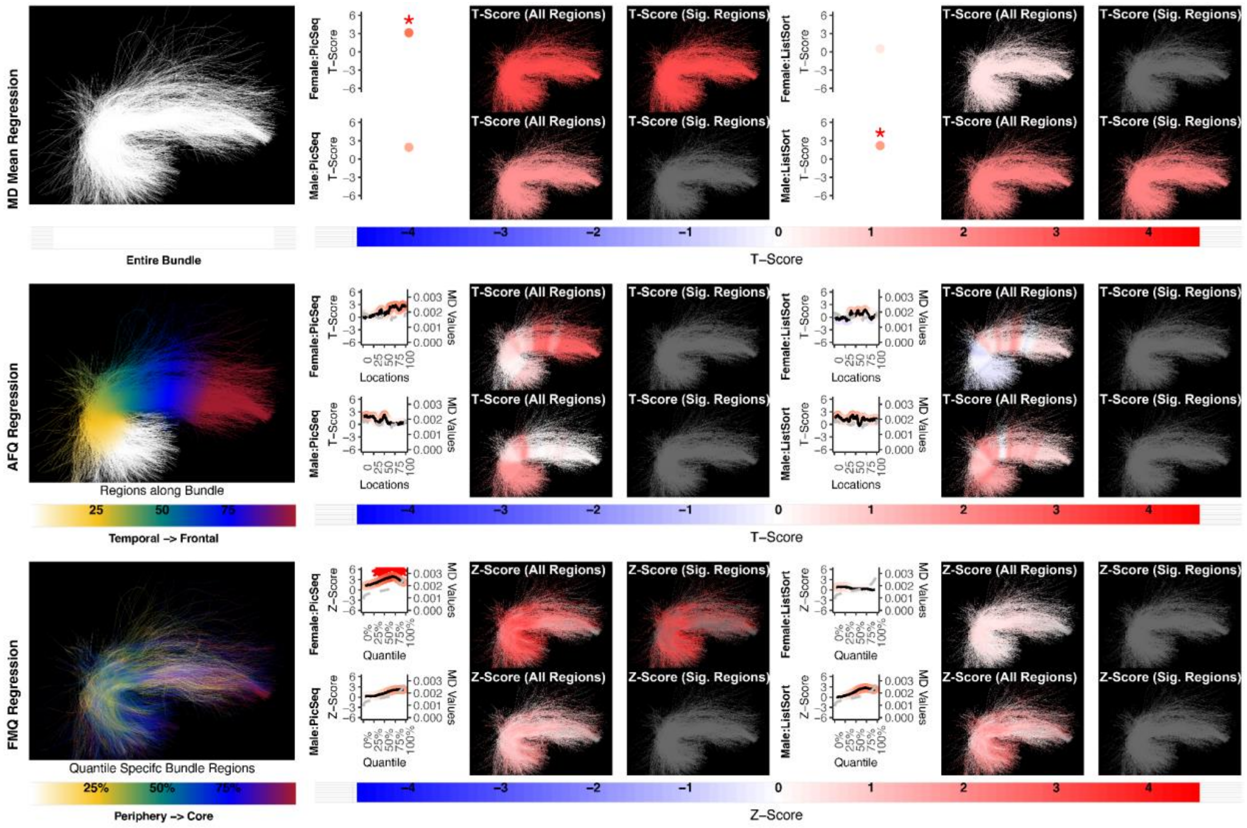

Figure S4. The association between UF right MD and memory performance. For each experiment, plots of Z- or T-scores (solid line) and FA (dashed line) are provided, with red asterisks indicating BH-FDR-corrected statistical significance. Visualizations of Z- and T-scores are provided.

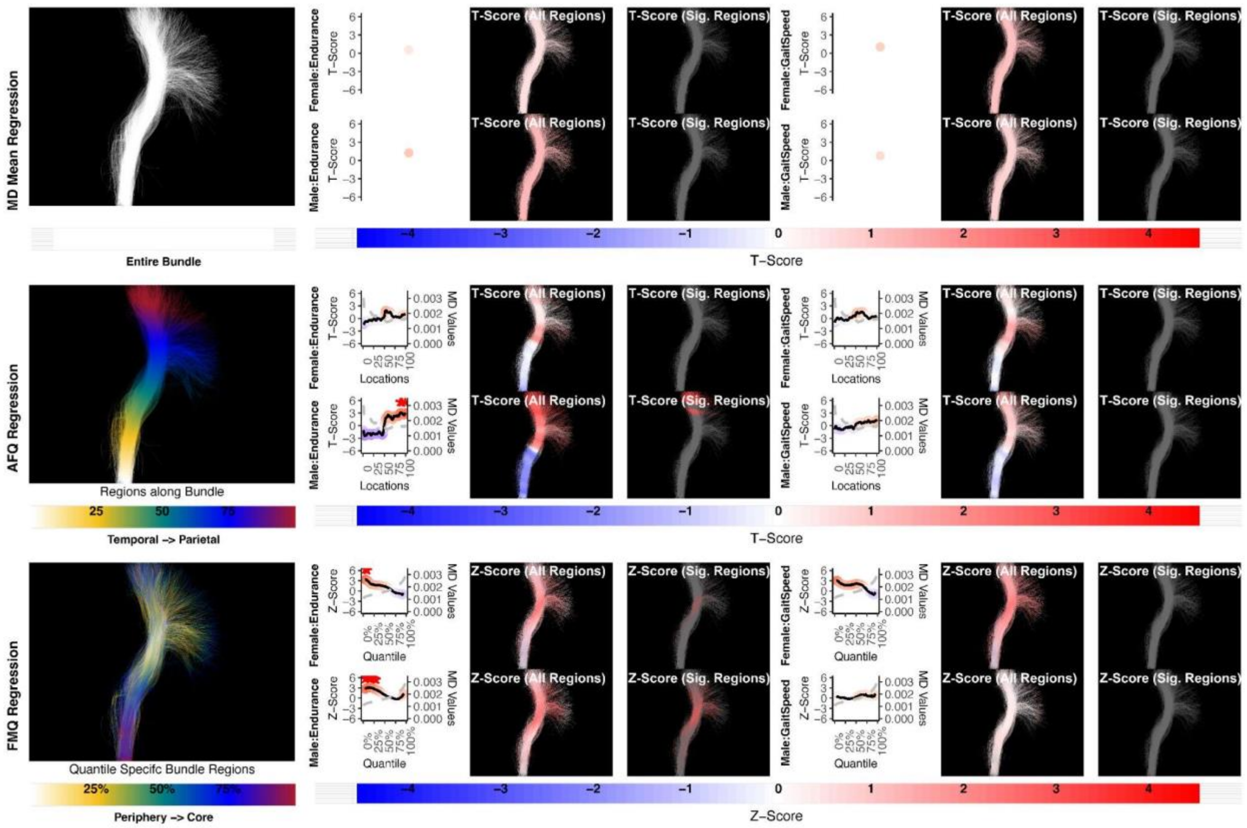

Figure S5. The association between CST left and motor performance. For each experiment, plots of Z- or T-scores (solid line) and FA (dashed line) are provided, with red asterisks indicating BH-FDR-corrected statistical significance. Visualizations of Z- and T-scores are provided.

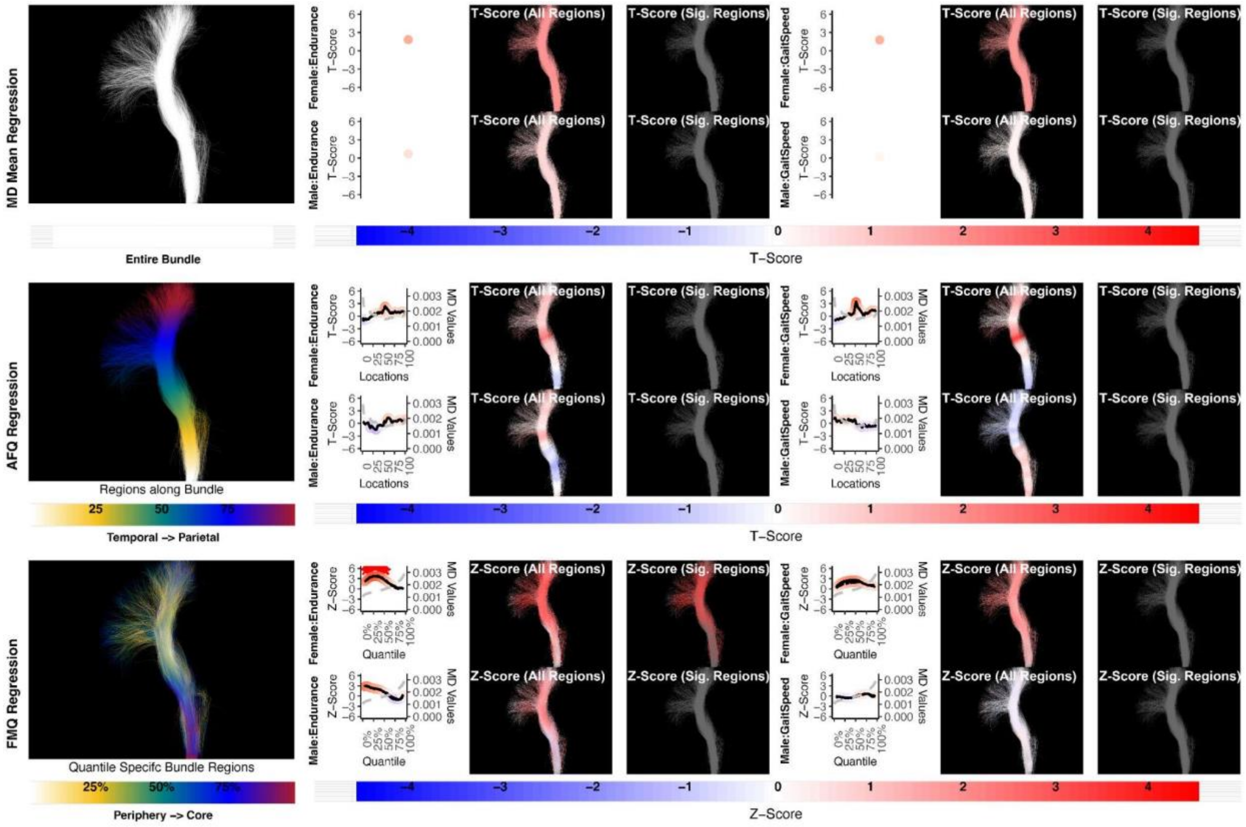

Figure S6. The association between CST right and motor performance. For each experiment, plots of Z- or T-scores (solid line) and FA (dashed line) are provided, with red asterisks indicating BH-FDR-corrected statistical significance. Visualizations of Z- and T-scores are provided.

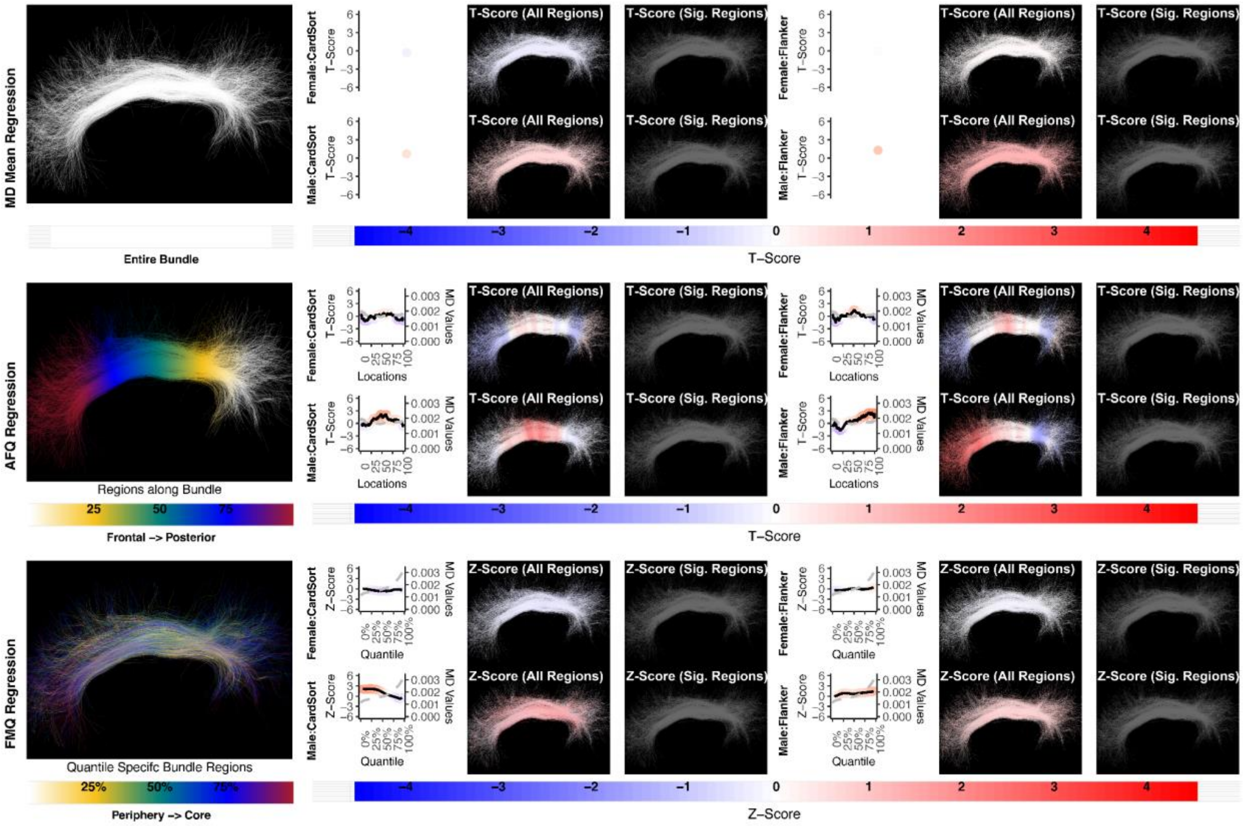

Figure S7. The association between CB left MD and Executive function. For each experiment, plots of Z- or T-scores (solid line) and FA (dashed line) are provided, with red asterisks indicating BH-FDR-corrected statistical significance. Visualizations of Z- and T-scores are provided.

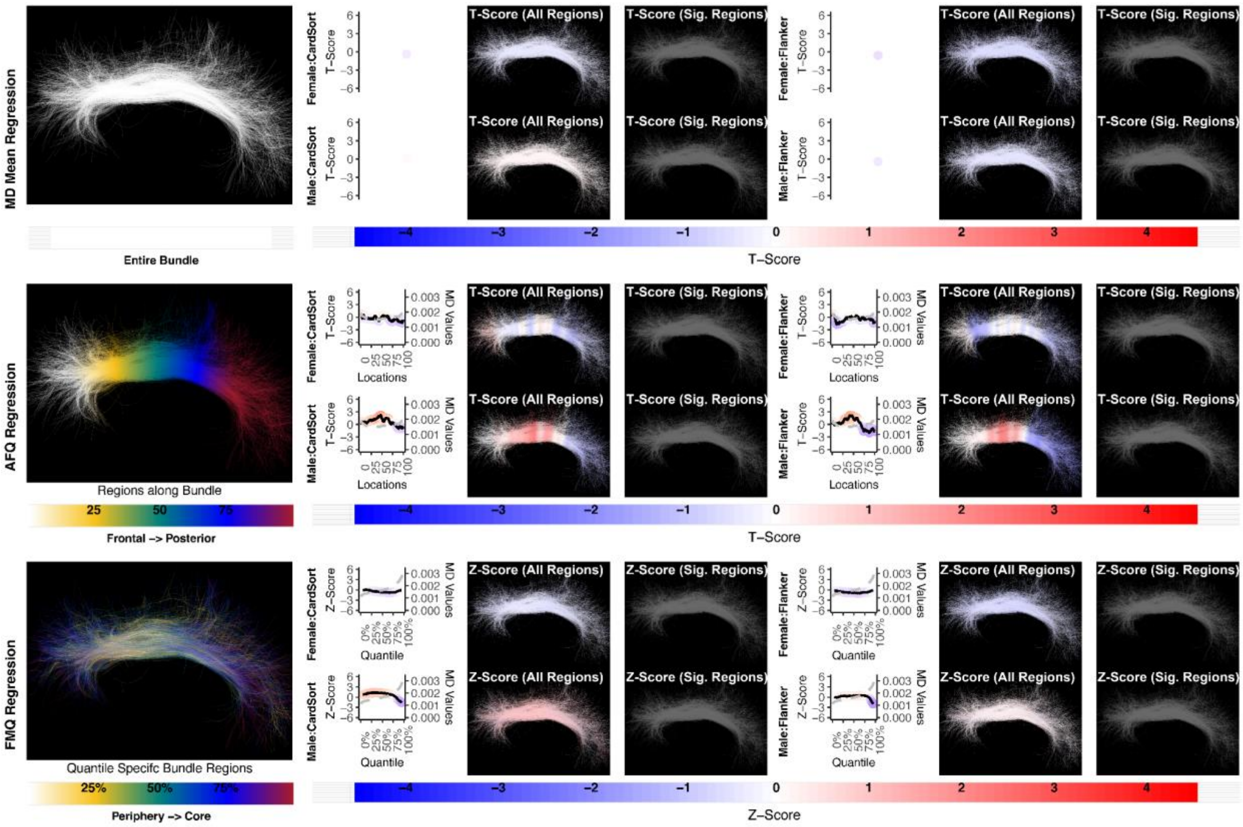

Figure S8. The association between CB right MD and Executive function. For each experiment, plots of Z- or T-scores (solid line) and FA (dashed line) are provided, with red asterisks indicating BH-FDR-corrected statistical significance. Visualizations of Z- and T-scores are provided.
